# Supplementary material for: Progress on Catastrophic Health Expenditure in China: Evidence from China Family Panel Studies (CFPS) 2010 to 2016
Source: Int J Environ Res Public Health. 2019 Nov 28;16(23):4775. doi: 10.3390/ijerph16234775 (PMC6926556; doi:10.3390/ijerph16234775)
Supplement: Supplementary file 1 [file ijerph-16-04775-s001.pdf]

Constructions of catastrophic health expenditure variables

Following previous studies by Ke Xu, our study defined catastrophic health expenditure (CHE) as an out-of-pocket payment for health care exceeded 40% of a household's capacity to pay.

Out-of-pocket (OOP) payment

Over the past 12 months, the total direct medical expenditure included costs for outpatient and inpatient care and other types of health care, excluding what was reimbursed or reimbursable but including what was paid by or borrowed from relatives.

Subsistence spending

After adjustment for standardized household size, we ranked the household spending on food as a percentage of total expenditure. Food expenditure was labeled as *food* in the Family Questionnaire Dataset from 2010 to 2016.

We calculated the equivalent household size and equalized food expenditures for each household as follows:

$$eqsize = householdsize^{0.56}$$

$$eqfood = \frac{food\ expenditures}{eqsize}$$

Subsistence spending was defined as the average household's standardized food expenditure as a share of total expenditure when the percentage was ranked between the 45th and 55th percentiles of the entire sample.

The subsistence spending of each household was calculated as the poverty line multiplied by standard household size.

$$pl(poverty\ line) = \frac{\sum weight \times eqfood}{\sum weight}$$

$$subspend = pl \times eqsize$$

Capacity to pay

Capacity to pay for the household was defined as the total household expenditure minus subsistence spending. Total household expenditure was labeled as *expense* in the Family Questionnaire Dataset from 2010 to 2016.

If a household's food expenditure was less than the subsistence spending, capacity to pay was defined as total expenditure minus food expenditure.

$$capacity\ to\ pay = expense - subspend\ if\ subspend \leq food$$

$$capacity\ to\ pay = expense - food\ if\ subspend > food$$

Households which didn't report their total expenditure, food expenditure or out-of-pocket

health expenditure were excluded from the analysis.

Catastrophic health expenditure

Catastrophic health expenditure was defined as an out-of-pocket payment for health care exceeded 40% of a household's capacity to pay.

$$CHE(Catastrophic\ health\ expenditure) = 1\ if\ \frac{oop}{capacity\ to\ pay} \geq 0.4$$

$$CHE(Catastrophic\ health\ expenditure) = 0\ if\ \frac{oop}{capacity\ to\ pay} < 0.4$$

Table S1. Trend of CHE in China calculated by alternative methods (OOP >10% or 25% of total household expenditure), 2010-2016.

Year of Interview	Mean Family Net Income [a]	Incidence of CHE (10% threshold, 95% CI) [b]	Incidence of CHE (25% threshold, 95% CI) [b]	Number of Households
2010	33069.51	30.35% (28.86%, 31.85%)	12.78% (11.81%, 13.76%)	12851
2012	36011.81	28.01% (26.64%, 29.38%)	11.25% (10.31%, 12.18%)	11477
2014	45705.77	26.78% (25.56%, 28.00%)	10.73% (9.95%, 11.51%)	13668
2016	54134.96	25.09% (24.06%, 26.13%)	9.96% (9.27%, 10.65%)	13884
Total	42253.73	27.84% (27.06%, 28.61%)	11.45% (10.96%, 11.94%)	51880

CHE: Catastrophic health expenditure. CI: Confidence interval. NA: Not available. [a] Prices in 2010 RMB Yuan. [b:] P < .001 for linear trends of the incidence of CHE across years.

Table S2. Trend of OOP budget share (OOP as % of household total expenditure) in China, 2010-2016.

Year of Interview	Mean Family Expenditure [a]	Mean OOP payment [a]	OOP budget share of household (95% CI) [b]	Number of Households
2010	30465.28	3117.11	10.73% (10.18%, 11.29%)	12851
2012	42851.30	3679.14	10.11% (9.60%, 10.61%)	11477
2014	59191.46	4374.88	10.46% (9.77%, 11.16%)	13668
2016	74943.63	5466.16	8.93% (8.58%, 9.28%)	13884
Total	52061.59	4117.05	10.06% (9.75%, 10.37%)	51880

OOP: Out-of-pocket. CI: Confidence interval. NA: Not available. [a] Prices in 2010 RMB Yuan. [b] P < .001 for linear trends of the incidence of CHE across years.

Figure S1. The population pyramids of the raw datasets (full sample, left) and analytic datasets (sample excluding the missing observations, right).

2010

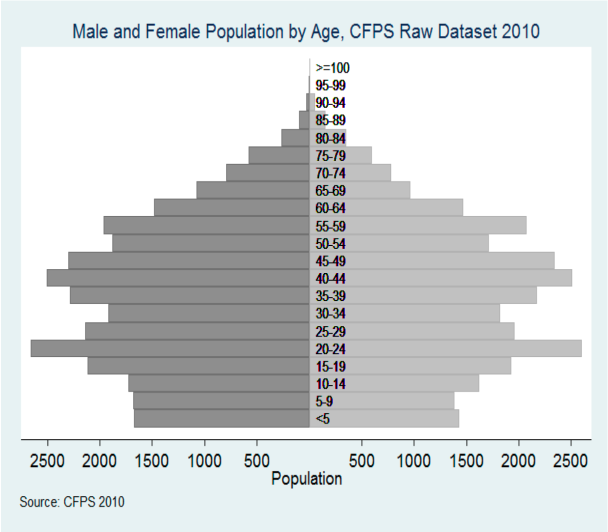

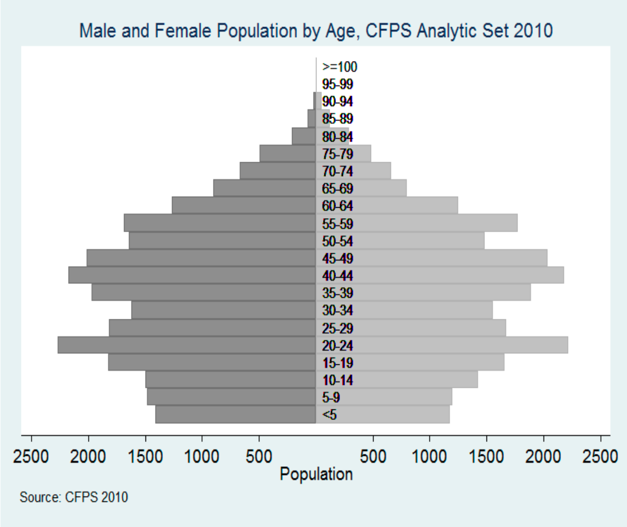

2012

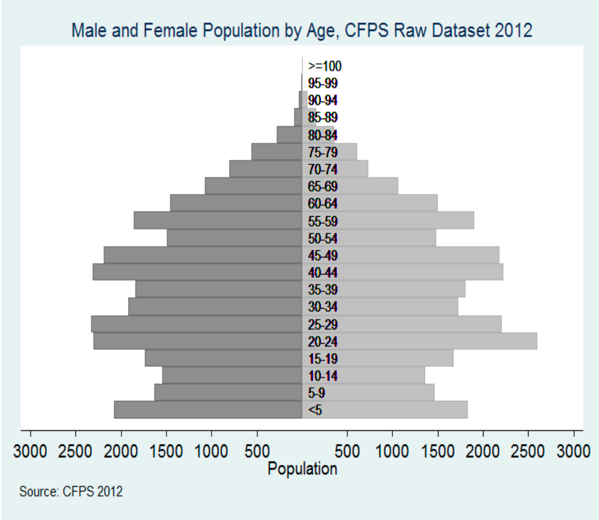

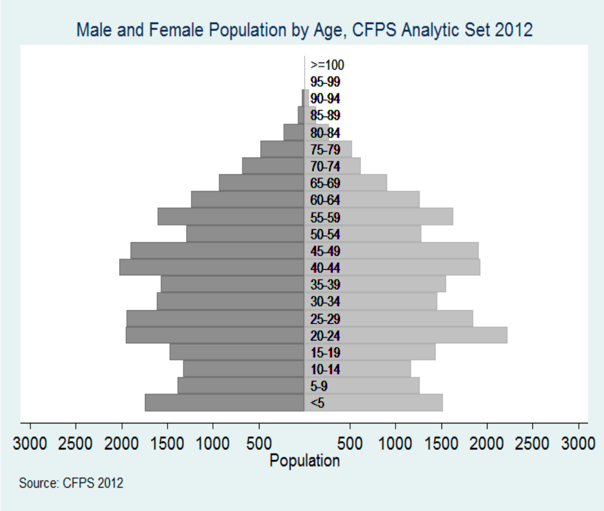

2014

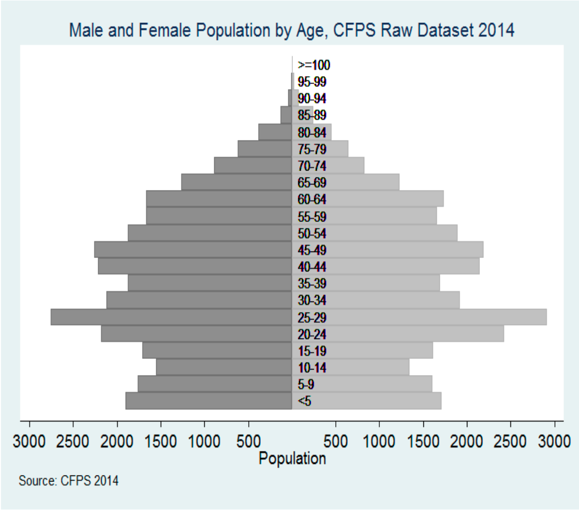
Male and Female Population by Age, CFPS Raw Dataset 2014
Population
Source: CFPS 2014

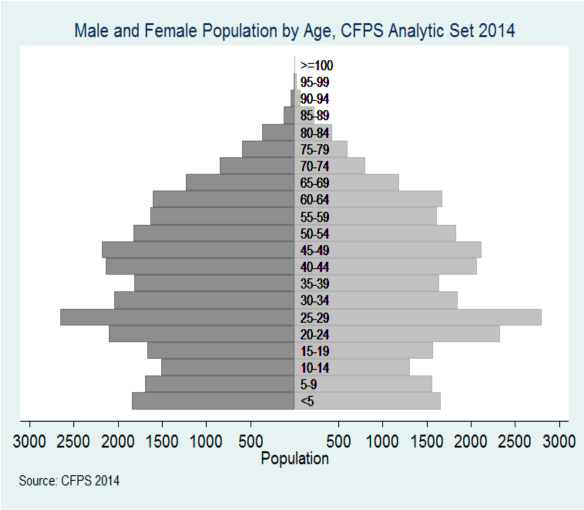
Male and Female Population by Age, CFPS Analytic Set 2014
Population
Source: CFPS 2014

2016

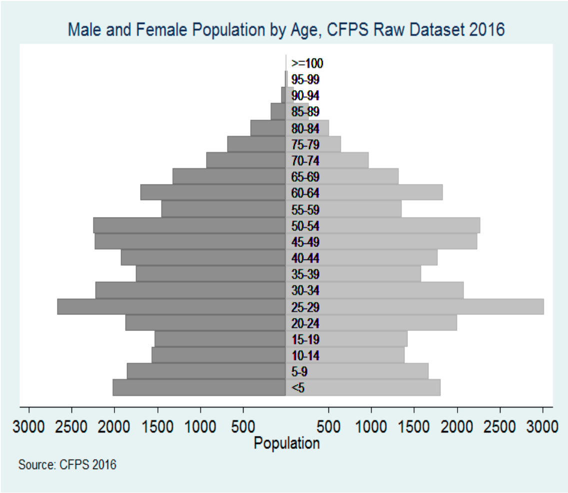
Male and Female Population by Age, CFPS Raw Dataset 2016
Population
Source: CFPS 2016

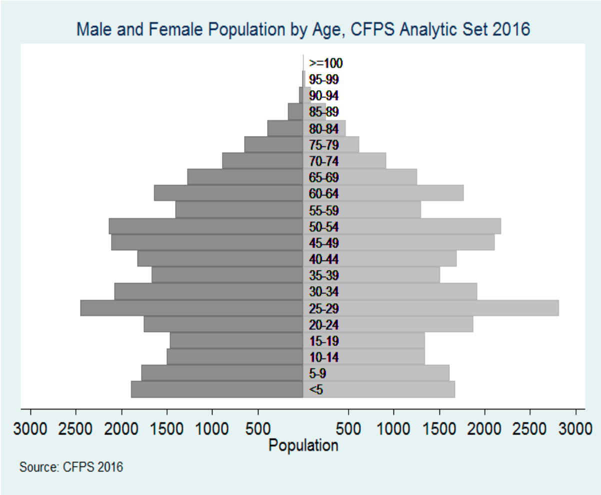
Male and Female Population by Age, CFPS Analytic Set 2016
Population
Source: CFPS 2016
